# Awareness on weaning diet among mothers of under five children in North Gujarat

**DOI:** 10.6026/97320630019032

**Published:** 2023-01-31

**Authors:** Mahalakshmi B, Vaikunthbhai Solanki Pujaben, Sivasubramanian N, Shaijo KJ

**Affiliations:** 1 Nootan College of Nursing, Sankalchand Patel University, Visnagar, Gujarat -384315, India

**Keywords:** Weaning diet, Mothers, Under five children, Awareness

## Abstract

Malnutrition caused by ineffective weaning techniques used during infancy and early childhood might affect cognitive and social development, school performance, and productivity in later life. The aim of the study was to evaluate the efficacy of education program in enhancing the awareness of mothers of under 5 children regarding weaning diet. The data was collected from 60 mothers of under 5 year's children. The existing awareness was assessed using a self-structured questionnaire. After pre intervention test, the mothers were provided with educational pamphlets and teaching programme with the help of pictures and charts. Benefits of complementary feeding, types of diet and its importance were included in the teaching program. The effectiveness of the teaching programme was assessed seven days after the start of the education programme. The mean score on the pre-test was 6.98, and the mean score on the post-test was 15.46. The average disparity was 8.48. The pre-test knowledge score's standard deviation was 3.85 and the post-test knowledge score's standard deviation was 4.23. The DF value was 59, the p value was 1.671, and the calculated "t" value is 24.98. At the 0.05 level of significance, the estimated "t" value (24.98) was higher than the table value (1.67) indicating that the education programme was successful in improving mothers' knowledge of weaning food.

## Background:

The World Health Organization (WHO) advises delaying supplemental feeding until when the infant is six months old because, up until then, nursing alone is sufficient to provide all of the baby's nutritional needs. Given that proper nutrition, capable of delivering sufficient nutritional quantity and quality, is required to support the growth and overall development in its maximum potential, complementary feeding is recognised as a significant physiological milestone in the life of the baby [1]. Additionally to ensuring an adequate diet in terms of nutrition, complementary feeding techniques have the potential to encourage the best eating-related habits and skills. To encourage and direct parents in using complementary feeding techniques, evidence-based guidelines for weaning diet must take into account the effects of this diet [2]. It is an important period in a baby's development since it coincides with the child's rapid growth as well as the emergence of dietary preferences, eating habits, and body weight in childhood, adolescence, and adulthood. As a result, the way a child is weaned may have an impact on that person's entire life [3]. Malnutrition caused by ineffective weaning techniques used during infancy and early childhood might affect cognitive and social development, school performance, and productivity in later life. They must be fed a diet that contains all the vitamins, minerals, and energy needed for regular growth as well as the nutrition and energy they need to be healthy and strong [4]. Many youngsters of weaning age are unable to grow well due to poor nutrition and disease. Poor weight gain or, in more severe cases, weight loss can be seen on the growth chart as a result. A major social determinant of the health of children has been discovered as the mother's educational position. Weaning is a challenging and potentially dangerous stage in an infant's development, and the success of the process is known to be influenced by the mother's education [5]. 75 breastfeeding moms who were visiting the paediatric outpatient department of the BV Hospital Bahawalpur with their infants participated in a cross-sectional descriptive study. It is suggested that mothers need to be taught on the value of weaning, the age at which weaning should occur, and the many kinds of infant weaning diets. It is important to underline the World Health Organization's recommended time frame as well as its benefits and drawbacks for early and delayed weaning [6]. It is of interest of the researchers to enhance the knowledge of Indian mothers of under 5 children regarding the benefits and importance of weaning diet.

## Methodology:

The present study was adopted an experimental research design. The aim of the study was to evaluate the efficacy of education program in enhancing the awareness of mothers of under 5 children regarding weaning diet. The data was collected from 60 mothers of under 5 year's children. Samples were selected from various villages of Mehsana District of Gujarat State, by purposive sampling method. The existing awareness was assessed using a self-structured questionnaire. After pre intervention test, the mothers were provided with educational pamphlets and teaching programme with the help of pictures and charts. Benefits of complementary feeding, types of diet and its importance were included in the teaching program. The effectiveness of the teaching programme was assessed seven days after the start of the education programme. The collected data was analyzed by various statistical methods such as mean, standard deviation and chi-square.

## Results:

**Figure 1:**
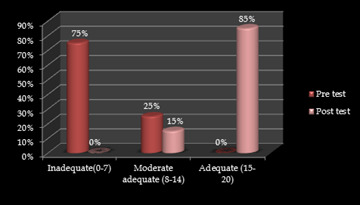
Cylinder diagram depicting percentage distribution of the samples according to the pre-test and post-test level of knowledge

Out of 60 subjects majority of sample 48.33% belongs to age of 27-30 and least 11.66% was in age above the 35 year. In terms of type of family, out of 60 subjects majority of sample 60% belongs to joint family and 40% was belongs to nuclear family. Majority of sample 40% belongs illiterate and least 10% was higher secondary. As regard to occupation of mothers, out of 60 subjects majority of sample 58.33% belongs to housewife and least 10% was had government job. 75% of sample had only one child under 5 years and least 5% had more than 4 children under 5 years.

(Figure 1) shows that prior to the administration of teaching programme, in the pre-test (75%) of the all sample had poor knowledge, 25% had moderate knowledge and no one had adequate knowledge. In the post test there was marked improvement in the knowledge of the sample with (15%) gainedmoderate knowledge and (85%) gained good knowledge.The data presented in Table 1 states that the mean of pre-test score was 6.98 and mean of post-test score was 15.46. The mean difference was 8.48. The standard deviation of pre-test knowledge score was 3.85 and standard deviation post-test knowledge score was 4.23. The calculated 't' value is 24.98, the DF value was 59 and p value was 1.671. The calculated 't' value (24.98) was greater than the table value (1.67) at 0.05 level of significance that shows the teaching programme was effective in increasing the knowledge of mothers regarding weaning diet.

## Discussion:

The study's objective was to assess the effectiveness of an education programme in raising mothers of children under the age of five's awareness of weaning diet. The examination of the data results showed that the educational programme was successful in raising mothers' knowledge and awareness of the weaning diet. An additional Lahore-based investigation that was undertaken backs up this conclusion. The purpose of the study was to create and assess the efficacy of a weaning teaching tool among mothers of infants between the ages of 6 and 24 months. The post-test results showed that weaning knowledge had dramatically increased. The study found that moms' knowledge of weaning can have a significant impact on the health of children who are weaning-age [7]. Another study, also done in Rewa, M.P., looked at the impact of structured education programmes on mothers of young children's awareness of weaning. The study's key finding was that the majority of new mothers had low to mediocre understanding of weaning foods. The study's designed teaching programme was discovered to be an efficient instrument for enhancing the mother's knowledge [8]. Another study modified the pre-experimental design of the one group pre-test and post-test approach using the mother's knowledge as the dependent variable and planned health education on weaning as the independent variable. The study was carried out in Hyderabad, A.P.'s urban slums of Rahmath and Sriram Nagar. There was a substantial difference in the post-test knowledge scores following the planned health education, indicating that exposure to the planned health education will result in an increase in knowledge [9]. Many more studies are available to support the effect of educational packages for improving knowledge and awareness among mothers of young children.

## Conclusion:

For a child to have healthy growth and development, adequate nutrition during the first year of life is a crucial requirement. The social standing of the family and the parents' educational attainment all has an impact on the success of supplemental feeding [10]. The current study aims to assess the effectiveness of educational programmes in raising mothers of children under the age of five's awareness of weaning diet. The study's findings showed that educational programmes were successful in raising mothers' knowledge and awareness of weaning diet.

## Figures and Tables

**Table 1:** Mean, S.D, Mean difference and 't' value of pre-test and post-test knowledge scores of effectiveness of structured teaching programme. DF= n-1 (60-1) =59

Parameter	Mean	Standard deviation	Mean difference	't' value	Table 't' value
Pre-test	6.98	3.85	8.48	24.98	1.671
Post-test	15.46	4.23			
